# Novel *Rickettsia* spp. in two common overwintering North American songbirds

**DOI:** 10.1080/22221751.2022.2140610

**Published:** 2022-11-11

**Authors:** Daniel J. Becker, Allison Byrd, Tara M. Smiley, Mariana Fernandes Marques, Julissa Villegas Nunez, Katherine M. Talbott, Jonathan W. Atwell, Dmitriy V. Volokhov, Ellen D. Ketterson, Alex E. Jahn, Kerry L. Clark

**Affiliations:** a Department of Biology, University of Oklahoma, Norman, OK, USA; b Department of Biology, Indiana University, Bloomington, IN, USA; c Environmental Resilience Institute, Indiana University, Bloomington, IN, USA; d Department of Ecology & Evolution, Stony Brook University, Stony Brook, NY USA; e Department of Public Health, University of North Florida, Jacksonville, FL, USA; f Center for Biologics Evaluation and Research, U.S. Food and Drug Administration, Silver Spring, MD, USA

**Keywords:** American robin, dark-eyed junco, rickettsiae, *borrelia*, *bartonella*, hemoplasmas, migration, arthropod-borne disease, stable isotopes

## Abstract

American robins and dark-eyed juncos migrate across North America and have been found to be competent hosts for some bacterial and viral pathogens, but their contributions to arthropod-borne diseases more broadly remain poorly characterized. Here, we sampled robins and juncos in multiple sites across North America for arthropod-borne bacterial pathogens of public health significance. We identified two novel *Rickettsia* spp. in one wintering migrant per bird species related to bellii, transitional, and spotted rickettsiae fever groups. Stable isotope analyses of feathers suggested spring migration of these common songbirds could disperse these novel rickettsiae hundreds-to-thousands of kilometers to host breeding grounds. Further work is needed to characterize zoonotic potential of these rickettsiae and host reservoir competence.

## Main text

Migratory birds play potentially important roles in shaping arthropod-borne disease risks, as they have the potential to disperse pathogens and vectors over large spatial scales. For pathogens with high public health burdens, such as *Borrelia burgdorferi* and spotted fever group (SFG) rickettsiae, migratory birds can disperse many infected vectors annually[1]. Many migratory birds are also competent hosts for these pathogens, as they can not only become infected and disperse pathogens and vectors but also transmit infection to naïve vectors after migration^[2,3]^.

American robins (*Turdus migratorius*) and dark-eyed juncos (*Junco hyemalis*) are important species for understanding bird migration and arthropod-borne disease in North America. They are competent reservoirs for some viral and bacterial pathogens (e.g. *B. burgdorferi*) and can have high ectoparasite intensities (e.g. of *Ixodes* ticks)[2,4]. Both species have diverse migratory behaviors, are widely distributed, and are common in suburban habitats[5,6], which could facilitate pathogen dispersal to areas of high human exposure to arthropod vectors. Yet the contribution of these common bird species to arthropod-borne bacterial disease remains poorly understood.

Here, we sampled robins and juncos in multiple sites across North America for select arthropod-borne pathogens of public health significance. We focused on bacterial pathogens for which these bird species are known to be competent (*Borrelia* spp.)[2], for which detections have occurred in other songbirds (*Rickettsia* spp.)[7], and that have either limited host range or have not yet been identified in birds (*Bartonella* spp. and hemoplasmas, respectively)[8].

Robins were sampled monthly in southern Indiana (2020–2021) as part of a longitudinal study of migratory behavior and infectious disease, while juncos were sampled in southern California (2006), Virginia’s Appalachian Mountains (2018, 2019), and northeastern Ohio (2019), spanning the diverse geographic range of this species. California and Ohio were sampled in the breeding season, while Virginia was sampled in winter[9]. We captured birds with mist nets, aged and sexed birds based on morphology[10], and applied USGS bands. Blood was collected using sterile needles and heparinized capillary tubes and stored in 96% ethanol or Longmire’s buffer at –20°C, on Whatman FTA cards at room temperature, or frozen directly at –20°C. We also collected the first secondary feather for stable isotope analyses and recorded ordinal fat score[10]. Sampling was approved by the Indiana University IACUC (06-242, 18-028), Federal Bird Banding Permit 20261, and state permits. Table S1 shows the sample sizes per site and month.

DNA was extracted from blood with Maxwell RSC Whole Blood DNA kits (Promega) or DNeasy 96 Blood and Tissue kits (Qiagen). We then used published PCR protocols to screen avian DNA for *Bartonella* spp. (partial *gltA* gene)*, Borrelia* spp. (partial 16S rRNA gene), and *Rickettsia* spp. (23S-5S rRNA intergenic spacer [ITS]). A subset of robins was also tested for hemoplasmas (partial 16S rRNA gene). Table S2 provides PCR primers and target amplification conditions.

Of 675 samples, we detected rickettsiae in one robin (0.26%, 1/391) and one junco (0.35%, 1/284), representing the first reports of rickettsiae in these bird species. No other target pathogens were detected (Table S1). These two positive amplicons were sequenced in both directions using the primers used for PCR. Both sequences (GenBank accessions ON773823.1 and ON773824.1) shared only 82.61% partial identity (with sequence coverage 60%) of the 23S and 5S rRNA flanking sequences of their ITS to one another, indicating distinct rickettsiae. We then used NCBI BLASTn to identify related rickettsiae 23S-5S rRNA ITS and type strain sequences, followed by MUSCLE for sequence alignment and MrBayes for phylogenetic analyses (run for 10,000,000 generations with the GTR+G+I model) via NGPhylogeny.fr[11,12].

**Figure 1.**
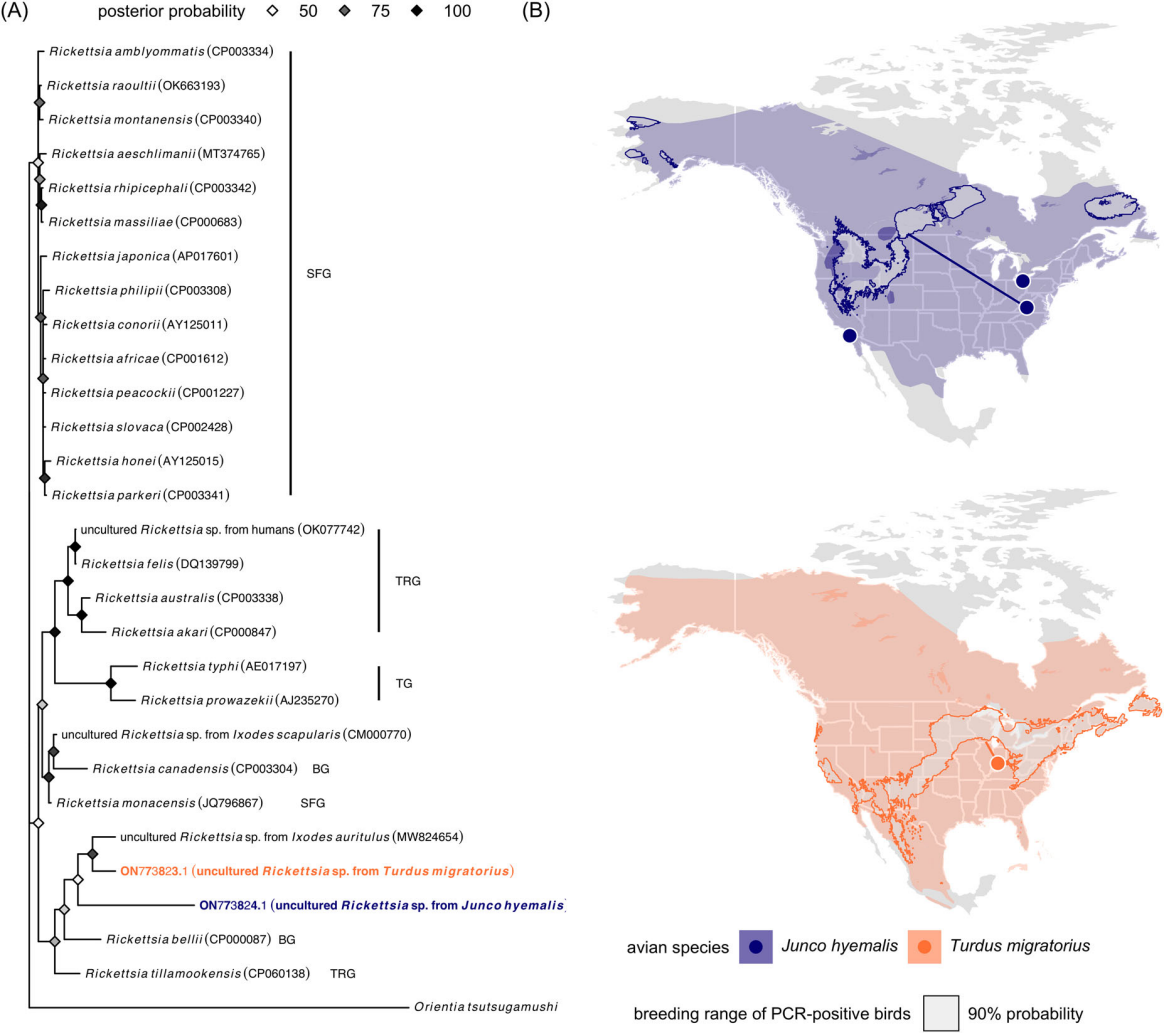
(A) Bayesian phylogeny of the novel *Rickettsia* spp. with closely related and reference rickettsiae sequences from GenBank, including those in the bellii group (BG), typhus group (TG), transitional group (TRG), and spotted fever group (SRG). *Orientia tsutsugamushi* (NZ_LYMT01000407) is used as an outgroup. Nodes are colored by posterior probability. (B) Field sites relative to the American robin and dark-eyed junco distribution with the estimated breeding origins of the two PCR-positive birds. Geographic assignments were performed using feather hydrogen of previously established known-origin juncos, robins, and other passerines (Fig. S1). Paths display the corresponding median migration distances, defined as kilometers between the winter capture site and median coordinates in the 90% probability breeding ground.

Both sequences had ≤91% identity to other *Rickettsia* spp. The robin sequence was partially related (83.85–91.01% identity with 91% sequence coverage) to uncultured rickettsiae from *Ixodes auritulus* in Argentina (MW824654), *Amblyomma americanum* and *Ixodes scapularis* in the USA (KJ796407 and KJ796403), humans in Ethiopia (MK693112) and India (OK077732–OK077742), and to *R. monacensis* (e.g. JQ796867), *R. tillamookensis* (CP060138), and *R. felis* (e.g. DQ139799). The junco sequence was also partially related (87.63–88.66% identity with 49% sequence coverage) to the above sequences, and the main internal part (146 bp) of this ITS sequence (without 23S and 5S rRNA flanking sequences) was unique with no identity to any rickettsiae ITS sequences in GenBank. These sequences thus belong to two novel but not yet cultured *Rickettsia* spp., and our 23S-5S ITS phylogeny suggested they are most similar to rickettsiae within the bellii group (BG), transitional group (TRG), and SFG[13] (Figure 1A).

Both the *Rickettsia*-positive robin and junco were adult females sampled during the winter (12/2020 and 11/2018, respectively). Most robins wintering in Indiana remain year-round or migrate elsewhere to breed (Jahn et al., unpublished). Wintering juncos in the Appalachians include migrant and resident subspecies[14], and we earlier determined this rickettsiae-positive junco to be migratory (*J. h. hyemalis*)[9]. Using feather hydrogen isotopes and geographic assignment models (Figure S1)[15], we estimated the most likely breeding site of the robin to be the Great Lakes (459 km median migration distance). In contrast, estimated breeding sites of the junco ranged from the western USA to Manitoba in Canada (2,377 km median migration distance; Figure 1B). Positive birds were thus short – or long-distance migrants, and both also had subcutaneous fat scores of zero, indicating that they had recently finished migration. Such results therefore suggest that these migratory songbirds could spread their novel rickettsiae to breeding grounds during spring migration, when they could then potentially infect naive vectors. Further study of these novel rickettsiae in these common birds and their arthropod vectors will be important to characterize the evolutionary history of these pathogens, whether these hosts are competent reservoirs, and their dispersal potential in relation to bird migration patterns.

## Supplementary Material

Supplemental Material
