# Supplementary material for: Novel Rickettsia spp. in two common overwintering North American songbirds
Source: Emerg Microbes Infect. 2022 Nov 11;11(1):2746–8. doi: 10.1080/22221751.2022.2140610 (PMC9662038; doi:10.1080/22221751.2022.2140610)
Supplement: Supplemental Material [file TEMI_A_2140610_SM5395.zip › Figure S1.docx]

**Figure S1.** Geographic assignments of rickettsiae-positive individuals, *Turdus migratorius* 1412-68667 (A,B) and *Junco hyemalis* 1881-71194 (C,D), based on the hydrogen isotopic value of flight feathers (for details about sample preparation, hydrogen equilibration, and isotopic analysis, see Wanamaker et al., 2020). Breeding location estimates presented in Figure 1 in the main text (B,D) reflect cells within the top 10% highest posterior probability based on Bayesian assignment (A,C). Geographic assignments were performed in R using the *assignR* package (Ma et al., 2020), using growing-season-precipitation (GSP; Bowen et al., 2005) and guild-specific (short-distance migrant, non-ground foraging) and species-specific resident calibration datasets for *T. migratorius* and *J. hyemalis*, respectively.  The calibration model for *T. migratorius* produced an intercept of -39.9, approximating the diet-to-tissue discrimination factor, and explained 69% of the isotopic variation in resident individuals.  The calibration models for *J. hyemalis* produced an intercept of -21.3, approximating the diet-to-tissue discrimination factor, and explained 48% of the isotopic variation in resident individuals. Seasonal ranges from IUCN (2019) are shown in light gray underneath estimates (species’ full range maps were used to construct geographic assignment maps).


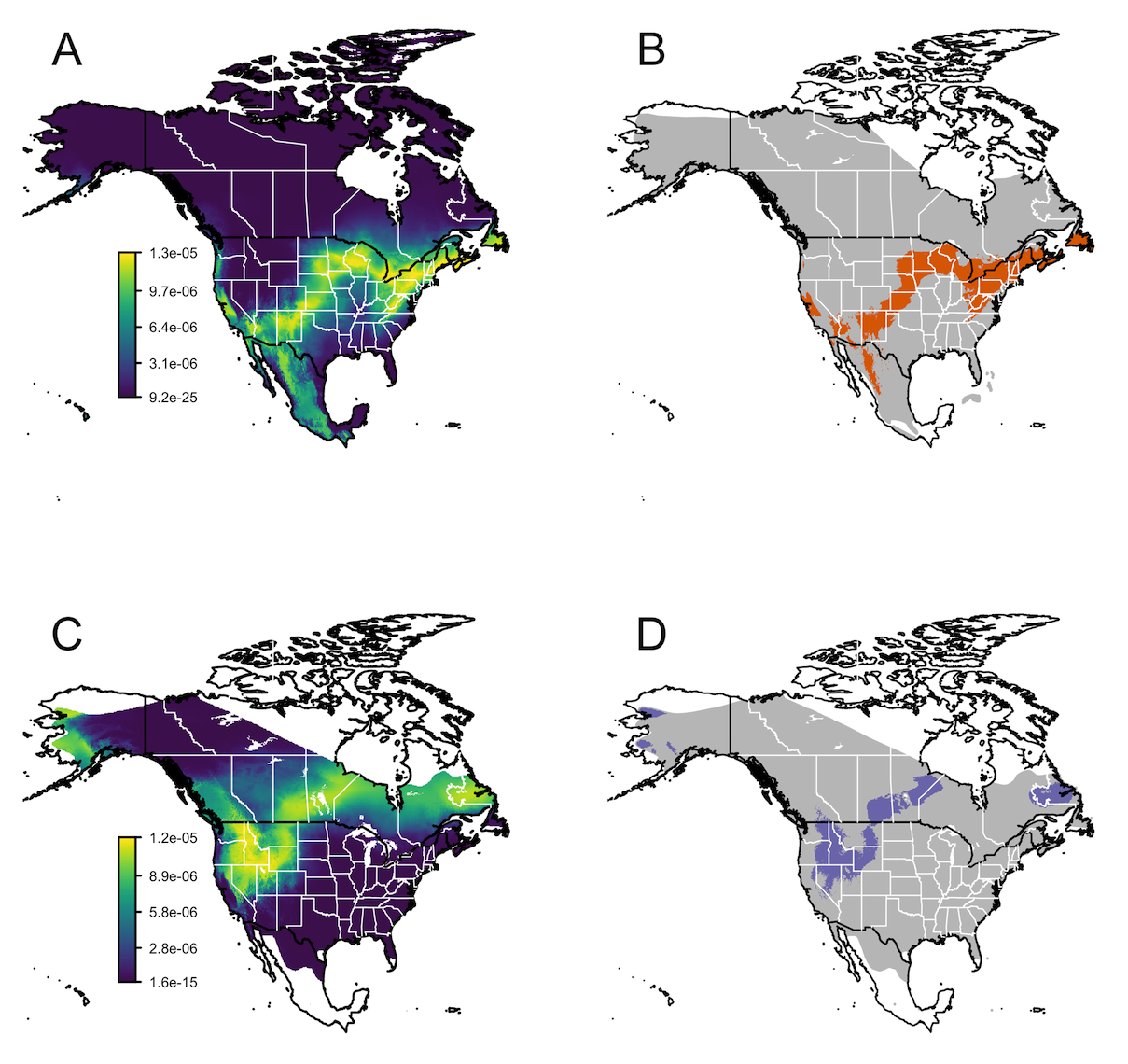


**References**

1. Bowen GJ, Wassenaar LI, Hobson KA. Global application of stable hydrogen and oxygen isotopes to wildlife forensics. *Oecologia*. 2005;143(3):337-48.
2. Wanamaker SM, Singh D, Byrd AJ, Smiley TM, Ketterson ED. Local adaptation from afar: migratory bird populations diverge in the initiation of reproductive timing while wintering in sympatry. *Biology Letters*. 2020;16(10):20200493.
3. Ma C, Vander Zanden HB, Wunder MB, Bowen GJ. assignR: An R package for isotope‐based geographic assignment. *Methods in Ecology and Evolution*. 2020;11(8):996-1001.
4. IUCN. 2019 The IUCN red list of threatened species. Version 2019–3. Retrieved from <http://www.iucnredlist.org>
