# Supplementary material for: Novel Rickettsia spp. in two common overwintering North American songbirds
Source: Emerg Microbes Infect. 2022 Nov 11;11(1):2746–8. doi: 10.1080/22221751.2022.2140610 (PMC9662038; doi:10.1080/22221751.2022.2140610)
Supplement: Supplemental Material [file TEMI_A_2140610_SM5395.zip › Table S1.docx]

**Table S1.** Sample sizes for robins and juncos per month, year, and region. All blood DNA samples were tested for *Borrelia* spp, *Rickettsia* spp., and *Bartonella* spp.; parentheticals indicate samples also tested for hemoplasmas. Asterisks indicate the single *Rickettsia*-positive sample per bird species.

| **Avian host** | **Date** | **Region** | **Number sampled** |
| --- | --- | --- | --- |
| *Turdus migratorius* | 1/2020 | Southern Indiana | 21 (8) |
| *Turdus migratorius* | 2/2020 | Southern Indiana | 15 (8) |
| *Turdus migratorius* | 3/2020 | Southern Indiana | 77 (41) |
| *Turdus migratorius* | 4/2020 | Southern Indiana | 67 (32) |
| *Turdus migratorius* | 5/2020 | Southern Indiana | 8 (7) |
| *Turdus migratorius* | 9/2020 | Southern Indiana | 18 |
| *Turdus migratorius* | 10/2020 | Southern Indiana | 20 |
| *Turdus migratorius* | 11/2020 | Southern Indiana | 25 |
| *Turdus migratorius* | 12/2020 | Southern Indiana | 24* |
| *Turdus migratorius* | 1/2021 | Southern Indiana | 21 |
| *Turdus migratorius* | 2/2022 | Southern Indiana | 1 |
| *Turdus migratorius* | 3/2022 | Southern Indiana | 18 |
| *Turdus migratorius* | 4/2022 | Southern Indiana | 37 |
| *Turdus migratorius* | 7/2021 | Southern Indiana | 5 |
| *Turdus migratorius* | 9/2021 | Southern Indiana | 28 |
| *Turdus migratorius* | 10/2021 | Southern Indiana | 6 |
| *Junco hyemalis* | 2/2006 | Southern California | 25 |
| *Junco hyemalis* | 3/2006 | Southern California | 54 |
| *Junco hyemalis* | 4/2006 | Southern California | 32 |
| *Junco hyemalis* | 5/2006 | Southern California | 25 |
| *Junco hyemalis* | 6/2006 | Southern California | 24 |
| *Junco hyemalis* | 7/2006 | Southern California | 9 |
| *Junco hyemalis* | 7/2019 | Northeastern Ohio | 20 |
| *Junco hyemalis* | 11/2018 | Appalachian Mountains | 65* |
| *Junco hyemalis* | 11/2019 | Appalachian Mountains | 30 |
