# Supplementary material for: Novel Rickettsia spp. in two common overwintering North American songbirds
Source: Emerg Microbes Infect. 2022 Nov 11;11(1):2746–8. doi: 10.1080/22221751.2022.2140610 (PMC9662038; doi:10.1080/22221751.2022.2140610)
Supplement: Supplemental Material [file TEMI_A_2140610_SM5395.zip › Table S2.docx]

**Table S2.** PCR primers and amplification parameters used in this study.

| **Primers** | **Sequence** | **Organism and gene^a^** | **Product size (bp)** | **Temperature (⁰C) / time (seconds)** | | | **Cycles** | **Ref** |
| --- | --- | --- | --- | --- | --- | --- | --- | --- |
|  |  |  |  | Denaturation | Annealing | Extension |  |  |
| Bart 443f | 5’-GCTATGTCTGCATTCTATCA-3’ | *Bartonella* *gltA* | 750 | 94/30 | 48/30 | 72/30 | 30 | 1 |
| Bart 1210r | 5’-GATCYTCAATCATTTCTTTCCA-3’ |  |  |  |  |  |  | 2 |
| BhCS 781p | 5’-GGGGACCAGCTCATGGTGG-3’ |  | 370 | 94/30 | 55/30 | 72/30 | 30 | 3 |
| BhCS 1137n | 5’-AATGCAAAAAGAACAGTAAACA-3’ |  |  |  |  |  |  | 3 |
| BSP 16S1A | 5’-CTAACGCTGGCAGTGCGTCTTAAGC-3’ | *Borrelia*  16S rRNA | 724 | 94/30 | 60/30 | 72/30 | 30 | 4 |
| BSP 16S1B | 5’-AGCGTCAGTCTTGACCCAGAAGTTC-3’ |  |  |  |  |  |  | 4 |
| BSP 16S2A | 5’-AGTCAAACGGGATGTAGCAATAC-3’ |  | ~657 | 94/30 | 55/30 | 72/30 | 30 | 4 |
| BSP 16S2B | 5’-GGTATTCTTTCTGATATCAACAG-3’ |  |  |  |  |  |  | 4 |
| RCK23/5-F | 5’-GATAGGTCRGRTGTGGAAGCAC-3’ | *Rickettsia* 23S-5S rRNA ITS | ~380 | 94/30 | 60/30 | 72/30 | 30 | 5 |
| RCK23/5-R | 5’-TCGGGAYGGGATCGTGTGTTTC-3’ |  |  |  |  |  |  | 5 |
| RCK23/5-NF | 5′-TGTGGAAGCACAGTAATGTGTG-3′ |  | ~350 | 94/30 | 55/30 | 72/30 | 30 | 6 |
| RCK23/5-NR | 5′-TCGTGTGTTTCACTCATGCT-3′ |  |  |  |  |  |  | 6 |
| UNI_16S_mycF | 5′-GGCCCATATTCCTACGGGAAGCAGCAGT-3′ | Hemotropic mycoplasma  16S rRNA | ~1000 | 95/300 | 60/60 | 72/60 | 50 | 7 |
| UNI_16S_mycR | 5′-TAGTTTGACGGGCGGTGTGTACAAGACCTG-3′ |  |  |  |  |  |  |  |

^a^*Bartonella* spp. *gltA*, citrate synthase gene; *Borrelia* spp. 16S rRNA gene; *Rickettsia* spp. 23S-5S rRNA intergenic spacer; hemotropic mycoplasma spp. 16S rRNA gene

**References**

1. Birtles RJ, Raoult D. Comparison of partial citrate synthase gene (*gltA*) sequences for phylogenetic analysis of *Bartonella* species. *International Journal of Systematic and Evolutionary Microbiology*. 1996;46(4):891-7.
2. Billeter SA, Osikowicz LM, Burns JE, Konde L, Gonzales BJ, Hu R, Kosoy MY. Molecular surveillance for *Bartonella*, *Borrelia*, and *Rickettsia* species in ticks from desert bighorn sheep (*Ovis canadensis*) and mule deer (*Odocoileus hemionus*) in Southern California, USA. *Journal of Wildlife Diseases*. 2018;54(1):161-4.
3. Norman AF, Regnery R, Jameson P, Greene C, Krause D. Differentiation of *Bartonella*-like isolates at the species level by PCR-restriction fragment length polymorphism in the citrate synthase gene. *Journal of Clinical Microbiology*. 1995;33(7):1797-803.
4. Richter D, Schlee DB, Matuschka FR. Relapsing fever–like spirochetes infecting European vector tick of Lyme disease agent. *Emerging Infectious Diseases*. 2003;9(6):697
5. Jado I, Escudero R, Gil H, Jiménez-Alonso MI, Sousa R, García-Pérez AL, Rodríguez-Vargas M, Lobo B, Anda P. Molecular method for identification of *Rickettsia* species in clinical and environmental samples. *Journal of Clinical Microbiology*. 2006;44(12):4572-6.
6. Kakumanu ML, Ponnusamy L, Sutton HT, Meshnick SR, Nicholson WL, Apperson CS. Development and validation of an improved PCR method using the 23S-5S intergenic spacer for detection of rickettsiae in *Dermacentor variabilis* ticks and tissue samples from humans and laboratory animals. *Journal of Clinical Microbiology*. 2016;54(4):972-9.
7. Volokhov DV, Norris T, Rios C, Davidson MK, Messick JB, Gulland FM, Chizhikov VE. Novel hemotrophic mycoplasma identified in naturally infected California sea lions (*Zalophus californianus*). *Veterinary Microbiology*. 2011;149(1-2):262-8.
